# Oral Health Care Behaviors and Periodontal Disease Among Older Adults with Diabetes Mellitus in Northern Thailand

**DOI:** 10.3390/geriatrics11050147

**Published:** 2026-10-16

**Authors:** Samart Kachiangrai, Parichat Ong-Artborirak, Katekaew Seangpraw

**Affiliations:** 1 School of Public Health, University of Phayao, Phayao 56000, Thailand; samarttodent@gmail.com; 2 Department of Research and Medical Innovation, Faculty of Medicine Vajira Hospital, Navamindradhiraj University, Bangkok 10300, Thailand; ong.artborirak@gmail.com

**Keywords:** oral health care behaviors, periodontal disease, older adults, Northern Thailand

## Abstract

**Introduction:** Periodontal disease is a major oral health problem among older adults with type 2 diabetes mellitus (T2DM). However, evidence regarding its association with oral health care behaviors in Northern Thailand is limited. This study aimed to assess oral health care behaviors and periodontitis and examine their associated factors among older adults with T2DM. **Methods:** A cross-sectional study was conducted among 369 older adults with T2DM in Mueang Phayao District, Northern Thailand. Seven subdistricts were selected using simple random sampling, after which eligible participants were recruited through convenience and snowball sampling. Data was collected using structured questionnaires and standardized oral examinations performed by dentists. **Results:** The mean oral health care behavior score was 29.51 out of 45, and periodontitis was present in 37.4% of the participants. Multivariable linear regression showed that oral health care behavior scores were significantly associated with oral health care knowledge scores (B = 1.052, *p* < 0.001), diabetes knowledge scores (B = 1.068, *p* < 0.001), FBS ≥ 126 mg/dL (B = −0.599, *p* = 0.027), and HbA1c ≥ 6.5% (B = −0.689, *p* = 0.016). Multivariable logistic regression showed higher odds of periodontitis among participants aged ≥70 years (AOR = 2.38, 95% CI: 1.45–3.89), with a diabetes duration of ≥11 years (AOR = 2.15, 95% CI: 1.21–3.82), who smoked (AOR = 12.62, 95% CI: 4.22–37.75), and with HbA1c ≥ 6.5% (AOR = 1.94, 95% CI: 1.11–3.41), while higher oral health care behavior scores were associated with lower odds (AOR = 0.88, 95% CI: 0.81–0.95). **Conclusions:** Advanced age, longer diabetes duration, smoking, and poor glycemic control were significantly associated with higher odds of periodontitis, whereas better oral health care behaviors were associated with lower odds. These findings support the integration of periodontal screening into diabetes care and the expansion of community-based oral health promotion for older adults with T2DM in primary care settings.

## 1. Introduction

Global demographic shifts toward population aging represent a major public health challenge in the twenty-first century. The United Nations projects that the number of individuals aged ≥60 years will increase from approximately 1.1 billion (14% of the global population) in 2022 to more than 2.1 billion (22%) by 2050 [1]. This demographic transition is accompanied by a substantial rise in the burden of noncommunicable diseases (NCDs), particularly diabetes mellitus, cardiovascular diseases, and chronic kidney disease, all of which adversely affect quality of life, functional capacity, and national health care expenditures [2,3]. Thailand has been classified as a complete aged society since 2021 and is projected to become a super-aged society by 2031, making the prevention and control of chronic diseases among older adults a high priority for the health system [4]. Aging is associated with multiple physiological changes, including immunosenescence, reduced salivary flow, polypharmacy, and declining self-care capacity, which collectively increase susceptibility to oral diseases, particularly periodontal disease [2,3].

Type 2 diabetes mellitus (T2DM) is one of the most prevalent noncommunicable diseases among older adults worldwide. The International Diabetes Federation estimates that the global number of people living with diabetes will increase from 537 million in 2021 to 783 million by 2045 [5], with a similar upward trend observed in Thailand, where 22.69% of older adults had diabetes in 2025. In Phayao Province, where the proportion of older adults exceeds the national average, diabetes remains among the most common chronic conditions, with more than 6000 older adults with diabetes residing in Mueang District [6]. This growing population faces an increased burden of both systemic and oral complications, particularly periodontal disease, tooth loss, and oral frailty, all of which impair quality of life and daily functioning [7,8,9]. Therefore, developing effective strategies for oral health promotion and disease prevention among older adults with diabetes is an important public health priority.

Periodontitis is a chronic inflammatory disease affecting the supporting tissues of the teeth and is a leading cause of tooth loss among older adults, with important implications for overall health and quality of life [10]. Its relationship with T2DM is bidirectional, as each condition may contribute to the severity and management challenges of the other [10,11,12]. The World Health Organization recognizes oral health as an essential component of general health and well-being [13]. In Thailand, findings from the 9th Thai National Oral Health Survey (2023) indicate that older adults retain an average of 19.6 teeth, while the prevalence of periodontal disease in the northern region is 58.4%, the highest reported among the regions of the country [14]. Evidence from systematic reviews further indicates that individuals with diabetes, particularly those with poor glycemic control, have a higher risk of periodontitis and tooth loss than the general population, with important consequences for oral health and quality of life [15]. In Phayao Province, 6135 older adults with diabetes were reported in Mueang District in 2025 [6]. Together, these findings highlight the importance of targeted oral health promotion and periodontal disease prevention among older adults with T2DM, particularly in Northern Thailand.

In Phayao Province, diabetes is among the most common chronic conditions affecting older adults, with 6135 older adults with diabetes reported in Mueang District in 2025, underscoring the need for targeted oral health promotion and periodontal disease prevention in this population [6]. Oral health behaviors are modifiable factors that play an important role in preventing periodontal disease [16]. Evidence from previous reviews indicates that appropriate toothbrushing, interdental cleaning, regular dental visits, glycemic control, and smoking avoidance are associated with a lower risk of periodontitis among older adults [15,17,18]. These behaviors are shaped by predisposing, enabling, and reinforcing factors [19], consistent with the PRECEDE–PROCEED model, which emphasizes the influence of individual, social, and environmental factors on health behaviors and provides a framework for developing context-specific health promotion interventions [20]. Although the relationship between diabetes and periodontitis has been extensively investigated internationally, evidence regarding the role of oral health behaviors in periodontitis among older adults with T2DM in Thailand remains limited, particularly in Northern Thailand, where the burden of periodontal disease is high. Therefore, this study aimed to examine the association between oral health behaviors and periodontitis among older adults with T2DM to provide evidence for contextually appropriate oral health promotion and disease prevention strategies. We hypothesized that older adults with T2DM who reported less favorable oral health behaviors would have a higher prevalence of periodontitis compared with those reporting more favorable behaviors.

## 2. Materials and Methods

### 2.1. Study Design and Study Period

A cross-sectional study was conducted between November 2025 and August 2026 among older adults with T2DM. Mueang Phayao District was selected as the study area because it has one of the largest older-adult populations in Northern Thailand, ranking among the top five districts, and its older population is projected to continue increasing. Mueang Phayao District comprises 15 subdistricts. Seven subdistricts, Mae Sai, Cham Pa Wai, Ban Tun, Ban Mai, Ban Sang, Ban Tam, and Ban Tom, were selected using simple random sampling by a lottery method. Data were collected at subdistrict health-promoting hospitals under the Phayao Provincial Administrative Organization, where routine primary healthcare and oral healthcare services are provided. Following the selection of the seven subdistricts, eligible participants were recruited using convenience sampling combined with snowball sampling. Recruitment information was disseminated through the participating subdistrict health-promoting hospitals to invite older adults with T2DM who met the eligibility criteria to participate. In addition, older adults in the participating communities who had been informed about the study were encouraged to inform other potentially eligible older adults about the study. All potentially eligible individuals were screened by the research team before enrollment. Because recruitment was conducted through community-based announcements and participant referral rather than individual invitations from a complete sampling frame, the number of eligible individuals approached, non-participants, and refusals was not recorded; therefore, a conventional response rate could not be calculated.

### 2.2. Study Setting, Population, and Sampling

The eligibility criteria for participants were as follows: (1) men or women aged 60 years or older; (2) residence in Mueang Phayao District for at least two years and registration in the civil registry; (3) registration in the Phayao Health Data Center in 2025 as a patient with T2DM and receipt of services from a subdistrict health-promoting hospital for at least nine months; (4) at least one natural tooth; (5) ability to communicate in the local language; and (6) provision of written informed consent. Individuals with cognitive impairment or acute illness on the data-collection date that prevented them from completing the questionnaire were excluded. The source population consisted of 6135 older adults with T2DM in Mueang Phayao District in 2025 and served as the basis for the sample size calculation. The sample size was determined using Daniel’s formula [21], assuming a 95% confidence level (Z = 1.96), a 5% margin of error (d = 0.05), and an a priori estimated prevalence of periodontitis of 36% (*p* = 0.36) [22], derived from previous literature among older adults with diabetes, with q = 1 − *p*. This prevalence estimate was used solely as a parameter for sample size determination and does not represent a finding from the current study. The minimum required sample size was 335 participants. To account for potential nonresponse or incomplete data, the sample size was increased by 10%, resulting in a target sample size of 369 participants.

### 2.3. Data Collection

Prior to field implementation, two qualified public health personnel, one dental assistant and one public health officer, were recruited as research assistants. They were selected primarily based on their prior experience in field data collection and health research, complemented by their native fluency in the northern local dialect and familiarity with the community’s cultural context to establish rapport and minimize potential information bias. Both assistants completed a standardized training session to ensure a comprehensive understanding of the study objectives, structured interviewing techniques, questionnaire administration, and research ethics regarding participant privacy and confidentiality. The research instruments were culturally adapted and translated into the local dialect to facilitate clear communication. Following formal coordination with the District Public Health Office, Sub-district Health Promoting Hospitals (SHPHs), and local community leaders, written informed consent was obtained from all participants.

Data collection was conducted sequentially: first, clinical oral health examinations were performed solely by a single licensed dentist (the principal investigator) with specialized clinical experience, thereby eliminating inter-examiner variability. These examinations strictly followed standard infection-control protocols and World Health Organization (WHO) oral health diagnostic criteria, utilizing standard dental units at the SHPHs or portable field dental chairs equipped with adequate artificial lighting, averaging approximately five minutes per participant. Subsequently, each participant underwent a 30 min face-to-face structured interview administered by the trained research assistants. To ensure data integrity, completeness, and quality control, the principal investigator personally reviewed and verified all completed questionnaires on-site immediately after each interview. Daily debriefing sessions were conducted with the research assistants to resolve any discrepancies or unclear responses. All fieldwork activities were carried out between 9:00 a.m. and 4:00 p.m. on scheduled days.

### 2.4. Research Instruments

A structured questionnaire was used as the primary instrument for data collection. Content validity was assessed by three experts in geriatric dentistry, family medicine, and health behavior. The items were evaluated using the Index of Objective Congruence (IOC), which yielded a value of 0.84. The questionnaire was developed based on the PRECEDE model [19] and comprised the following predisposing factors: (1) demographic and clinical characteristics, including sex, age, educational level, occupation, average monthly income (THB), duration of diabetes (years), self-reported comorbidity status (yes = presence of at least one comorbidity; no = no comorbidity), fasting blood glucose (FBS) (mg/dL), glycated hemoglobin (HbA1c) (%), diabetes medication adherence, smoking status (Current smoker, Non-smoker), and alcohol consumption. (2) Social support was assessed using a 10-item questionnaire. Each item had three response options: “yes,” “no,” and “uncertain.” A response of “yes” was scored as 1, whereas responses of “no” and “uncertain” were scored as 0. The total score ranged from 0 to 10, with higher scores indicating higher levels of social support. For descriptive purposes, total scores were categorized as low (0–5), moderate (6–7), or high (8–10). (3) Oral health care knowledge was adapted from relevant studies and contextualized for community-dwelling older adults in rural settings [23]. The instrument comprised 10 items. Example items include “Dry mouth in older adults is a contributing factor to dental caries, gingivitis, and periodontitis” and “Bleeding gums are an early sign of gingivitis and periodontitis.” Response options were “Yes,” “No,” and “Do not know.” (4) Diabetes knowledge consisted of 10 items adapted from previous studies to ensure suitability for older adults living in rural communities [23,24]. Example items include “Diabetes increases the risk of gingivitis and dental caries among older adults compared with those without diabetes” and “Individuals with diabetes often experience dry mouth, which increases the risk of dental caries.”

For the knowledge questionnaire, participants selected a single response for each item. Each correct answer was assigned 1 point, whereas incorrect or “do not know” responses received 0 points. The total score (ranging from 0 to 10) was categorized into three levels based on the percentage of the maximum possible score: low (<60%, 0–5 points), moderate (60–79%, 6–7 points), and high (≥80%, 8–10 points). (5) Oral health care behaviors were assessed using a 15-item questionnaire adapted from previous research [23]. Participants reported the frequency of each behavior during the preceding week, selecting one response per item. Responses were scored as “regularly” (5–7 times/week, 3 points), “sometimes” (2–4 times/week, 2 points), and “never” (0–1 times/week, 1 point). Total scores ranged from 15 to 45 points and were categorized into three levels based on the percentage of the maximum possible score: low (<60%, <27 points), moderate (60–79%, 27–35 points), and high (≥80%, 36–45 points). These scoring criteria and cut-off thresholds have been used and validated in previous studies. The overall questionnaire was pilot tested among 30 older adults with similar characteristics residing in the community. Reliability testing demonstrated that the oral health care knowledge and diabetes knowledge scales had Kuder–Richardson Formula 20 (KR-20) coefficients of 0.79 and 0.86, respectively. The oral health care behavior scale had a Cronbach’s alpha coefficient of 0.85. The complete English-translated version of the entire questionnaire, including all items for knowledge, behavior, and social support scales, is provided in Supplementary Materials. Enabling factors included dental service utilization, which was assessed using a structured multiple-choice questionnaire. Reinforcing factors comprised caregiver-related variables measured using a checklist format. Social support was evaluated using a 10-item scale with three response options: “agree,” “uncertain,” and “disagree.” Participants were instructed to select one response per item. Total scores were categorized into three levels: low, moderate, and high.

The Periodontal Screening and Recording (PSR) index was used to evaluate periodontal health. In accordance with the oral health care guidelines for patients with diabetes developed for dental and public health personnel [25], the oral cavity was divided into six sextants and evaluated using a WHO periodontal probe [26,27,28]. Before data collection, the examiner underwent training and calibration to ensure standardized assessment of periodontal conditions according to the PSR criteria. All periodontal examinations were performed by a single dentist who was a member of the research team. The same participants were re-examined after the initial examination to assess intra-examiner reliability. Cohen’s kappa coefficient was 0.85, indicating almost perfect agreement. Because only one examiner performed all periodontal examinations, inter-examiner reliability was not assessed. PSR scores were classified as follows: Code 0, healthy periodontal tissues with no signs of gingival inflammation; Code 1, bleeding on probing; Code 2, presence of supra- or subgingival calculus and/or plaque retention factors detected during probing; Code 3, moderate periodontitis with a probing pocket depth of 4–5.5 mm; and Code 4, severe periodontitis with a probing pocket depth ≥ 6 mm. An asterisk (*) was used to indicate clinical abnormalities, such as gingival abscesses, tooth mobility, or furcation involvement. Sextants with no teeth or fewer than two teeth were recorded as X. For the purposes of this study, participants were categorized according to their highest PSR code as follows: normal periodontal status (code 0), gingivitis (codes 1–2), and periodontitis (codes 3–4) [27]. Because PSR is a screening rather than a definitive diagnostic tool, these categories represent screening-based periodontal status and should not be interpreted as confirmed clinical diagnoses. Individuals with abnormal PSR findings should undergo a comprehensive periodontal examination for definitive diagnosis [27].

To assess glycemic status, blood samples were collected to measure FBS and HbA1c levels. Participants were instructed to fast for at least 12 h before blood collection (from 20:00 on the preceding day) and were scheduled for venipuncture the following morning at 07:00 at subdistrict health-promoting hospitals within the study area. Blood samples (5 mL) were obtained by a registered nurse and sent for analysis at the Phayao Hospital laboratory. Laboratory results were reported through the HOSxP information system of Phayao Hospital, and participants provided informed consent for the use of their laboratory data in this study.

### 2.5. Statistical Analysis

All data were analyzed using IBM SPSS Statistics version 26.0 (IBM, Armonk, NY, USA). Descriptive statistics, including frequencies (n), percentages, means, and standard deviations (SDs), were used to summarize participant characteristics and key study variables. Differences in mean oral health care behavior scores across participant characteristics and periodontal status were assessed using independent-samples *t*-tests or one-way ANOVA, with Welch’s correction applied when homogeneity of variance was violated. Pearson’s correlation coefficient (r) was used to assess relationships among continuous variables, including age, FBS, HbA1c, oral health care knowledge, diabetes knowledge, social support, and oral health care behavior scores. Associations between periodontitis and participant characteristics were examined using the chi-square test. Multiple linear and logistic regression analyses were performed to identify factors associated with oral health care behavior scores and periodontitis, respectively. Candidate covariates were selected based on the study’s conceptual framework, the previous literature, and clinical relevance. Variables with *p*-values < 0.20 in the initial analyses were considered for inclusion in the respective full models to avoid excluding potentially relevant variables at the screening stage. The models were then refined by sequentially removing variables that were not statistically significant (*p* ≥ 0.05), while retaining variables considered clinically important or potentially confounding based on prior evidence. The results were interpreted as adjusted associations rather than causal effects. Because only measured variables were included in the models, residual confounding from unmeasured or incompletely measured factors could not be excluded. Both unstandardized (B) and standardized (β) regression coefficients were reported for the linear regression model, whereas odds ratios (ORs) with 95% confidence intervals (CIs) were reported for the binary logistic regression model. Multicollinearity was assessed using the variance inflation factor (VIF), and model assumptions were evaluated using residual diagnostics for the linear regression model.

## 3. Results

### 3.1. Oral Health Care Behavior Scores, Periodontal Status, and Participant Characteristics

The final sample comprised 369 older adults with diabetes mellitus in Northern Thailand. The mean oral health care behavior score was 29.51 (SD = 3.45; range, 17–42), with 234 participants (63.4%) classified as having a moderate level of oral health care behaviors. Based on the highest PSR code recorded, 46 participants (12.5%) had a screening-based normal periodontal status, 185 (50.1%) had gingivitis, and 138 (37.4%) had periodontitis. The mean oral health care behavior scores differed significantly according to the periodontal health status (*p* < 0.001, Table 1).

The majority of participants were women (67.8%), with a mean age of 68.47 years (SD = 5.94). Most had an educational level of primary school or below (85.9%), more than half were employed (54.7%), and 81.8% had an average monthly income of no more than 5000 baht. The mean duration of diabetes was 10.24 years (SD = 7.72), and most adhered to their diabetes medication (86.4%). The mean FBS was 136.24 mg/dL (SD = 33.73), and the mean HbA1c was 7.25% (SD = 1.25). Comorbidities were present in 89.7% of participants, with dyslipidemia (76.7%) and hypertension (70.2%) being the most common conditions. Most did not smoke (93.0%), while 71.8% did not consume alcohol. Approximately 36.9% had never received dental services, while almost three-quarters had a caregiver (74.0%). The mean scores for oral health care knowledge, diabetes knowledge, and social support were 6.02 (SD = 1.33), 6.31 (SD = 1.24), and 5.64 (SD = 1.70), respectively (Table 2).

### 3.2. Factors Associated with Oral Health Care Behavior Scores

Mean oral health care behavior scores differed significantly among participants with different periodontal health status groups (*p* < 0.001, Table 1). Significant differences were observed across several factors, including FBS, HbA1c, oral health care knowledge, diabetes knowledge, social support, and patterns of dental service utilization (all *p* < 0.05; Table 2). When analyzed as continuous variables, oral health care behavior scores were significantly correlated with oral health care knowledge scores (r = 0.687), diabetes knowledge scores (r = 0.674), social support scores (r = 0.280), FBS levels (r = −0.259), and HbA1c levels (r = −0.251) (all *p* < 0.001; Table 3).

Variables with *p*-values < 0.20 in the previous analysis were included in the multiple linear regression model, consisting of income level, adherence to prescribed diabetes medication, dental service utilization, social support score, oral health care knowledge score, diabetes knowledge score, FBS, and HbA1c. In the final model, oral health care knowledge score (B = 1.052, *p* < 0.001), diabetes knowledge score (B = 1.068, *p* < 0.001), FBS ≥ 126 mg/dL (B = −0.599, *p* = 0.027), and HbA1c ≥ 6.5% (B = −0.689, *p* = 0.016) were significantly associated with oral health care behavior scores (adjusted R^2^ = 0.588; Table 4). The maximum VIF in the final model was 1.68, indicating no evidence of multicollinearity, and standardized residuals were approximately normally distributed.

### 3.3. Factors Associated with Periodontitis

Chi-square tests revealed significant associations between periodontitis and several factors, including age group, duration of diabetes, FBS, HbA1c, smoking, oral health care knowledge, diabetes knowledge, and social support (all *p* < 0.05; Table 2). Variables with *p*-values < 0.20 in the previous analysis were included in the multiple logistic regression model, consisting of age group, education level, income level, duration of diabetes, adherence to prescribed diabetes medication, smoking, dental service utilization, having a primary caregiver, social support score, oral health care knowledge score, diabetes knowledge score, oral health care behavior score, FBS, and HbA1c. A sensitivity analysis was performed by excluding current smokers (n = 26) to assess the robustness of the primary multivariable findings. The analysis included 343 participants. The direction, magnitude, and statistical significance of the main associations remained consistent with those of the primary analysis: age ≥70 years (OR = 2.46, 95% CI: 1.50–4.05), diabetes duration ≥11 years versus ≤5 years (OR = 2.05, 95% CI: 1.14–3.66), and HbA1c ≥ 6.5% (OR = 1.81, 95% CI: 1.02–3.23) remained associated with higher odds of periodontitis, whereas higher oral health care behavior scores remained associated with lower odds of periodontitis (OR = 0.88, 95% CI: 0.81–0.95). The sensitivity model yielded a Nagelkerke R^2^ of 0.205 (Table 5).

## 4. Discussion

In this community-based cross-sectional study, the findings indicate that older adults with T2DM who demonstrated good oral self-care, higher levels of diabetes-related knowledge, and adequate social support were significantly more likely to exhibit better oral health behaviors. Concurrently, oral examinations revealed that periodontal disease was highly prevalent, with most participants presenting with gingivitis and periodontitis rather than healthy periodontal status. These findings indicate an association between oral health behaviors and periodontal status [28]. This relationship may be explained by behavioral theory, whereby health behaviors are influenced by the interaction of knowledge, beliefs, enabling factors, and reinforcing factors, as described in the PRECEDE–PROCEED model [19]. Individuals are more likely to initiate and sustain appropriate health behaviors when they possess adequate skills and resources and receive support from family or their social networks. This is consistent with evidence showing that improved oral health behaviors are associated with a reduced risk of periodontitis among individuals with chronic diseases [29]. Among older adults, periodontal disease and tooth loss are also associated with diabetes and poor glycemic control, indicating that oral health care is an integral component of chronic disease management [7,30].

Correlation analyses further demonstrated that oral health behaviors among older adults with diabetes were significantly associated with both cognitive factors and clinical outcomes. Specifically, oral health behavior scores were positively correlated with oral health literacy, diabetes-related knowledge, and social support, and negatively correlated with FBS and HbA1c levels. These findings suggest that oral health behaviors extend past hygiene practices and are closely linked to glycemic control [30]. Participants also exhibited knowledge gaps in important areas, including the impact of periodontitis on tooth loss and the role of xerostomia in increasing the risk of dental caries, gingivitis, and periodontitis. This indicates that insufficient knowledge may hinder the adoption of appropriate oral health behaviors. Consistent with a recent systematic review, oral health education and support from health care providers can improve knowledge, promote positive oral health behaviors, and potentially improve glycemic control in individuals with diabetes [31].

An interesting finding was that blood glucose control, as measured by both FBS and HbA1c, was significantly associated with oral health care behavior scores; those whose biochemical values were within the controlled range tended to have better oral health care behavior. Oral health behavior scores also varied significantly according to levels of oral health literacy, diabetes knowledge, social support, and patterns of dental service utilization. In addition, age, duration of diabetes, smoking status, and both knowledge- and support-related factors were significantly associated with periodontitis. These findings indicate that oral health behaviors among older adults with diabetes are not determined by a single factor but arise from complex interactions among health status, cognitive factors, and the social environment [32]. Interpreted within the PRECEDE–PROCEED framework, the results suggest that knowledge and beliefs function as predisposing factors, while access to resources, utilization of dental services, and social support from family and community act as enabling and reinforcing factors that facilitate sustained oral health behaviors [19]. In older adults with chronic conditions, oral self-care is often influenced by both their understanding of diabetes and their ability to implement appropriate practices in daily life [32]. This is consistent with prior studies showing that oral health behaviors and the use of preventive dental services among individuals with diabetes remain suboptimal and that environmental and access-related factors are significantly associated with these behaviors [33]. Similarly, the present study, guided by this framework, demonstrates that oral health behaviors are associated with predisposing factors (knowledge and attitudes), enabling factors (access to resources and health services), and reinforcing factors (support from family, community, and health care providers), all of which contribute to the adoption and maintenance of appropriate oral health practices [29].

In addition, regarding periodontal outcomes, increasing age, longer duration of diabetes, elevated FBS and HbA1c levels, and smoking were significantly associated with periodontitis. These findings are consistent with pathophysiological evidence indicating that poorly controlled diabetes promotes inflammatory processes in periodontal tissues and accelerates periodontal destruction [8]. Furthermore, systematic reviews have indicated that inadequate plaque control and poor oral hygiene practices are associated with poorer periodontal health and may contribute to the progression of periodontal disease [34], whereas regular toothbrushing and routine dental visits reduce disease risk [35]. The present findings are also consistent with evidence from Sweden demonstrating that periodontitis in individuals with diabetes is associated with poor glycemic control, with greater disease severity observed among those with suboptimal metabolic control [17]. Similarly, a study from Bangladesh suggests that both diabetes self-care behaviors and oral health practices should be jointly considered in the prevention and control of periodontitis [32].

Further analysis indicates that oral health behaviors among older adults with diabetes are significantly associated with oral health knowledge, diabetes-related knowledge, and social support, while showing significant inverse associations with FBS and HbA1c levels. These findings are consistent with Pender’s Health Promotion Model, which posits that health behaviors are influenced by individual perceptions and experiences in interaction with interpersonal and environmental influences, thereby facilitating the adoption and maintenance of health-promoting behaviors [36]. In this context, predisposing, enabling, and reinforcing factors were associated with appropriate oral health behaviors, which may in turn be related to better glycemic control [19,33].

Mechanistically, appropriate oral health behaviors may reduce dental plaque accumulation, gingival inflammation, and the severity of periodontitis, all of which are associated with glycemic control. Recent evidence indicates that regular toothbrushing and interdental cleaning are associated with more favorable glucose profiles, including lower fasting glucose levels and improved markers of diabetes control [37]. These findings are consistent with prior studies demonstrating that oral care behaviors and oral hygiene are closely related to both periodontitis and glycemic control in individuals with diabetes [30]. Systematic reviews among older adults with diabetes further indicate that enabling factors, particularly access to dental services, support from health care providers, and self-care knowledge, are significantly associated with oral health behaviors. Interventions focusing on education and skill development may therefore enhance self-care practices and contribute to better glycemic outcomes [9,31]. Moreover, accumulating evidence suggests that advanced age, poor glycemic control, and inadequate oral health behaviors are significantly associated with both the risk and severity of periodontitis [7,18,32].

Multivariable logistic regression analysis identified age group, duration of diabetes, smoking status, oral health behavior scores, and HbA1c levels as significant predictors of periodontitis, with the model explaining 27.0% of the variance (Nagelkerke R^2^ = 0.270). These findings are consistent with the established bidirectional relationship between diabetes and periodontitis and indicate the particular vulnerability of older adults with diabetes. At the item level, participants demonstrated misconceptions regarding the effects of smoking on periodontal health and the impact of consuming sugary or sticky foods between meals on oral disease risk. In addition, suboptimal oral health practices were observed, including irregular toothbrushing after waking and before bedtime, as well as the consumption of erosive foods and beverages. These findings reflect persistent deficits in both knowledge and behavior that warrant targeted interventions. The results can also be interpreted within behavioral theory, which emphasizes the role of knowledge and individual factors in determining and sustaining health behaviors [36,38]. Increasing age and longer duration of diabetes may reflect cumulative physiological decline and prolonged exposure to metabolic dysregulation, leading to increased vulnerability of periodontal tissues and heightened inflammatory responses [7]. Smoking acts as an important risk factor that exacerbates disease severity, whereas appropriate oral health behaviors can reduce plaque accumulation and mitigate the risk of periodontitis. These findings are consistent with prior studies demonstrating that behavioral factors, together with glycemic control, play an important role in the development and progression of periodontitis in individuals with diabetes [8,19,28]. Elevated HbA1c levels reflect poor glycemic control and are associated with chronic inflammation and increased severity of periodontitis. Evidence from systematic reviews and longitudinal studies further supports the association of diabetes and poor glycemic control with a substantially increased risk and progression of periodontal disease [11,17]. Importantly, this study found that good oral health behaviors were significantly associated with a reduced likelihood of periodontitis, emphasizing the importance of consistent self-care practices, such as proper toothbrushing, effective oral hygiene, and avoidance of risk behaviors, in preventing periodontal disease among older adults with diabetes.

This study has several limitations. First, its cross-sectional design precludes causal inference and does not establish the temporal sequence between oral health behaviors and periodontal status. Reverse causality is possible because existing periodontal symptoms may have influenced participants’ oral health behaviors or their reporting of these behaviors. Therefore, the observed associations should not be interpreted as causal effects. Residual confounding also cannot be excluded because some relevant factors may not have been measured or included in the multivariable models, as reflected by the moderate Nagelkerke *R*^2^, suggesting that other factors may contribute to the observed variation in periodontitis. Second, convenience and snowball sampling were used to recruit individual participants, while the inclusion criteria were restricted to individuals receiving continuous health care services. This may have introduced selection bias, as participants who volunteered or were referred to by others may have differed from eligible older adults who did not participate. Because individual-level information on non-participants was unavailable, the extent of selection bias could not be quantitatively assessed. Although the study areas were selected using simple random sampling from 15 subdistricts, individual participants were recruited only from these selected areas. Therefore, the findings may not be fully generalizable to all older adults with T2DM in Mueang Phayao District or to other populations and settings. Third, some data were self-reported and may be subject to recall and reporting bias. In particular, comorbidity status was obtained through interviewer-administered self-report and classified only as present or absent. Detailed information on the type, severity, duration, treatment, and medication used for individual comorbid conditions was not collected; therefore, the associations of specific systemic conditions or their treatments with periodontal health could not be evaluated. Fourth, only a small proportion of participants reported current smoking. This limited number may have resulted in sparse cells and less stable regression estimates for smoking-related associations. A sensitivity analysis excluding current smokers showed that the main findings remained consistent with the primary analysis; however, smoking-related findings should be interpreted with caution and confirmed in larger studies.

Finally, periodontal status and behavioral variables were assessed at a single time point, limiting the ability to capture temporal changes. Future studies should use prospective cohort designs to clarify the temporal relationships between oral health behaviors and periodontitis among older adults with diabetes. Intervention studies evaluating the effects of oral health education, behavioral modification, and social support on glycemic control and periodontal outcomes are also warranted. Research across diverse settings and populations would further improve generalizability and inform public health policy.

## 5. Conclusions

This study demonstrates that oral health behaviors, oral health knowledge, diabetes-related knowledge, and social support play important roles in the oral health of older adults with T2DM. Individuals with greater knowledge and better self-care practices combined with adequate social support were likelier to exhibit favorable oral health behaviors and had a lower risk of periodontitis. Conversely, older age, longer duration of diabetes, smoking, and poor glycemic control were significantly associated with an increased risk of periodontitis. In summary, these findings indicate that periodontal disease prevention in older adults with diabetes should integrate multiple strategies: health education to improve knowledge, behavioral interventions to promote appropriate oral health practices, reduction in risk behaviors, and sustained glycemic control. Furthermore, family support, engagement of health care providers, and equitable access to dental services are essential components that should be incorporated into health promotion planning for this population.

## Figures and Tables

**Table 1:** Oral health care behavior scores and periodontal status of the participants (n = 369).

Variable	n (%)	Oral Health Care Behavior	
		Mean ± SD	Min–Max
Oral health care behavior		29.51 ± 3.45	17–42
Low level (scores of 15–26)	115 (31.2)		
Medium level (scores of 27–35)	234 (63.4)		
High level (scores of 36–45)	20 (5.4)		
Periodontal health status			
Normal (PSR code 0)	46 (12.5)	34.33 ± 2.55	30–42
Gingivitis (PSR codes 1–2)	185 (50.1)	29.16 ± 2.91	23–39
Periodontitis (codes 3–4)	138 (37.4)	28.37 ± 3.03	17–34
		*p*-value < 0.001 ^a^	

Note: ^a^ One-way ANOVA.

**Table 2:** Oral health care behavior scores and periodontitis by participant characteristics.

Factors	n (%)	Behavior Scores		Periodontitis	
		Mean ± SD	*p*-Value	Yes	*p*-Value
Predisposing factors					
Gender			0.590 ^a^		0.421 ^c^
Male	119 (32.2)	29.47 ± 3.66		40.3%	
Female	250 (67.8)	29.52 ± 3.35		36.0%	
Age group			0.270 ^a^		<0.001 ^c^
60–69 years	214 (58.0)	29.79 ± 3.63		29.9%	
≥70 years	155 (42.0)	29.11 ± 3.16		47.7%	
Education level			0.849 ^a^		0.092 ^c^
Primary and below	317 (85.9)	29.48 ± 3.42		39.1%	
Above primary	52 (14.1)	29.67 ± 3.65		26.9%	
Occupation			0.993 ^a^		0.738 ^c^
Unemployed	167 (45.3)	29.37 ± 3.42		38.3%	
Employed	202 (54.7)	29.62 ± 3.48		36.6%	
Average monthly income			0.152 ^a^		0.158 ^c^
≤5000 THB	302 (81.8)	29.34 ± 3.30		39.1%	
>5000 THB	67 (18.2)	30.27 ± 4.00		29.9%	
Duration of diabetes			0.297 ^b^		<0.001 ^c^
1–5 years	127 (34.4)	29.76 ± 3.54		29.9%	
6–10 years	117 (31.7)	29.65 ± 3.53		28.2%	
≥11 years	125 (33.9)	29.12 ± 3.27		53.6%	
Comorbidities			0.653 ^a^		0.527 ^c^
No	38 (10.3)	29.08 ± 3.50		42.1%	
Yes	331 (89.7)	29.56 ± 3.45		36.9%	
FBS			0.001 ^a^		<0.001 ^c^
<126 mg/dL	160 (43.3)	30.73 ± 3.73		26.9%	
≥126 mg/dL	209 (56.6)	28.57 ± 2.90		45.5%	
HbA1c			<0.001 ^a^		<0.001 ^c^
<6.5%	118 (32.0)	30.96 ± 3.89		24.6%	
≥6.5%	251 (68.0)	28.82 ± 3.00		43.4%	
Medication adherence			0.080 ^a^		0.140 ^c^
Daily adherence	319 (86.4)	29.41 ± 3.34		38.9%	
Non-adherence	50 (13.6)	30.14 ± 4.09		28.0%	
Smoking status			0.792 ^a^		<0.001 ^c^
Current smoker	26 (7.0)	28.08 ± 3.42		80.8%	
Non-smoker (never, former)	343 (93.0)	29.62 ± 3.43		34.1%	
Alcohol consumption			0.525 ^a^		0.615 ^c^
Yes	104 (28.2)	30.03 ± 3.73		39.4%	
No	265 (71.8)	29.30 ± 3.32		36.6%	
Oral health knowledge			<0.001 ^b^		<0.001 ^c^
Low (0–5 scores)	210 (56.9)	27.51 ± 2.67		43.3%	
Moderate (6–7 scores)	139 (37.7)	31.61 ± 1.97		33.8%	
High (8–10 scores)	20 (5.4)	35.80 ± 2.48		0%	
Diabetes knowledge			<0.001 ^b^		<0.001 ^c^
Low (0–5 scores)	179 (48.5)	27.08 ± 2.40		46.7%	
Moderate (6–7 scores)	154 (41.7)	31.12 ± 2.16		37.0%	
High (8–10 scores)	36 (9.8)	34.67 ± 2.54		2.8%	
Enabling factors					
Dental service utilization			0.025 ^b^		0.153 ^c^
Never	136 (36.9)	28.95 ± 3.49		43.4%	
Last visit > 1 year ago	166 (45.0)	29.64 ± 3.38		32.5%	
At least once a year	67 (18.2)	30.30 ± 3.40		37.3%	
Reinforcing factors					
Caregiver			0.385 ^a^		0.135 ^c^
No	96 (26.0)	29.13 ± 3.71		43.7%	
Yes	273 (74.0)	29.64 ± 3.35		35.2%	
Social support			0.001 ^b^		0.040 ^c^
Low (0–5 scores)	248 (67.2)	29.06 ± 3.27		40.7%	
Moderate (6–7 scores)	74 (20.1)	30.11 ± 3.49		36.5%	
High (8–10 scores)	47 (12.7)	30.91 ± 3.85		21.3%	

Note: ^a^ Independent-Samples *t* test; ^b^ One-Way ANOVA; ^c^ Chi-Square test.

**Table 3:** Pearson correlation coefficients among age, fasting blood sugar (FBS), glycated hemoglobin (HbA1c), oral health care knowledge, diabetes knowledge, social support, and oral health care behaviors.

Variable	(1)	(2)	(3)	(4)	(5)	(6)	(7)
(1) Age (years)							
(2) FBS (mg/dL)	−0.055						
(3) HbA1c (%)	−0.059	0.684 **					
(4) Oral health care knowledge (scores)	−0.091	−0.178 **	−0.131 *				
(5) Diabetes knowledge (scores)	−0.119 *	−0.175 **	−0.129 *	0.624 **			
(6) Social support (scores)	−0.029	−0.095	−0.057	0.256 **	0.240 **		
(7) Oral health care behaviors (scores)	−0.069	−0.259 **	−0.251 **	0.687 **	0.674 **	0.280 **	

Notes: * Significance at the 0.05 level (2-tailed). ** Significance at the 0.01 level (2-tailed).

**Table 4:** Factors associated with oral health care behavior scores: multiple linear regression analysis.

Variable	Full Model				Final Model			
	B	β	95%CI for B	*p*-Value	B	β	95%CI for B	*p*-Value
Income (>5000 THB)	0.413	0.046	−0.181, 1.007	0.172				
Medication adherence (non-adherence)	0.407	0.040	−0.263, 1.076	0.233				
Dental service utilization ^a^ Last visit > 1 year ago At least once a year	0.040 0.175	0.006 0.020	−0.474, 0.555 −0.491, 0.605	0.877 0.605				
Social support (score) ^b^	0.107	0.053	−0.360, 0.250	0.141				
Oral health knowledge (score) ^b^	1.026	0.397	0.803, 1.249	<0.001	1.052	0.407	0.831, 1.272	<0.001
Diabetes knowledge (score) ^b^	1.038	0.374	0.801, 1.274	<0.001	1.068	0.385	0.834, 1.303	<0.001
FBS (≥126 mg/dL)	−0.598	−0.086	−1.129, −0.067	0.027	−0.599	−0.086	−1.128, −0.069	0.027
HbA1c (≥6.5%)	−0.598	−0.081	−1.161, −0.035	0.037	−0.689	−0.093	−1.246, −0.132	0.016
	Adjusted R^2^ = 0.591				Adjusted R^2^ = 0.588			

Notes: ^a^ Reference group = never; ^b^ continuous data.

**Table 5:** Factors associated with periodontitis: multiple logistic regression analysis.

Variable	Full Model			Final Model		
	OR	95%CI	*p*-Value	OR	95%CI	*p*-Value
Age group (≥70 years)	2.335	1.380, 3.951	0.002	2.375	1.448, 3.893	0.001
Education level (Above primary)	0.423	0.186, 0.966	0.041			
Income (>5000 THB)	0.705	0.350, 1.420	0.328			
Duration of diabetes ^a^						
6–10 years	0.594	0.311, 1.133	0.114	0.705	0.380, 1.306	0.267
≥11 years	2.099	1.146, 3.844	0.016	2.147	1.206, 3.823	0.009
Medication adherence (non-adherence)	0.661	0.298, 1.470	0.310			
Smoking status (Current smoker)	16.545	5.059, 54.111	<0.001	12.619	4.218, 37.747	<0.001
Dental service utilization ^b^						
Last visit > 1 year ago	0.670	0.383, 1.171	0.160			
At least once a year	0.843	0.404, 1.761	0.650			
Caregiver (yes)	0.723	0.398, 1.314	0.288			
Social support (score) ^c^	0.969	0.830, 1.130	0.685			
Oral health knowledge (score) ^c^	1.194	0.915, 1.559	0.192			
Diabetes knowledge (score) ^c^	0.913	0.685, 1.217	0.535			
Oral health care behaviors (score) ^c^	0.874	0.778, 0.982	0.023	0.878	0.813, 0.949	0.001
FBS (≥126 mg/dL)	1.691	0.962, 2.974	0.068			
HbA1c (≥6.5%)	1.476	0.785, 2.774	0.227	1.943	1.107, 3.411	0.021
	Nagelkerke R^2^ = 0.318			Nagelkerke R^2^ = 0.270		

Notes: ^a^ Reference group = 1–5 years; ^b^ reference group = never; ^c^ continuous data.

## Data Availability

The data presented in this study are available on request from the corresponding author. The data are not publicly available due to privacy and ethical restrictions, as it contains sensitive personal and health-related information.

## Supplementary Materials

The following supporting information can be downloaded at https://www.mdpi.com/article/10.3390/geriatrics11050147/s1, Questionnaire.

## Abbreviations

The following abbreviations are used in this manuscript:

T2DM	Type 2 diabetes mellitus
NCDs	Non-communicable diseases
FBS	Fasting blood sugar
HbA1c	Glycated hemoglobin
PSR	Periodontal Screening and Recording
PRECEDE	Predisposing, Reinforcing, and Enabling Constructs in Educational/Ecological Diagnosis and Evaluation
PROCEED	Policy, Regulatory, and Organizational Constructs in Educational and Environmental Development
