# Supplementary material for: Oral Health Care Behaviors and Periodontal Disease Among Older Adults with Diabetes Mellitus in Northern Thailand
Source: Geriatrics (Basel). 2026 Oct 16;11(5):147. doi: 10.3390/geriatrics11050147 (PMC13704405; doi:10.3390/geriatrics11050147)
Supplement: Supplementary file 1 [file geriatrics-11-00147-s001.zip › geriatrics-4575165-supplementary.pdf]

Questionnaire

Part 1. Demographic and Health Characteristics

Note: Please place a ✓ mark in the box or fill in the blank with the information that best reflects your actual circumstances.

General Characteristics of the Participant

1. Gender

    0. Male 1. female

2. Age ________ year

3. Educational Level

    1. Primary and below
    2. Above primary

3. Occupation

    0. Unemployed
    1. Employed

6. Average monthly income

    1. ≤ 5,000 THB
    2. > 5,000 THB

7. Duration of diabetes

    1. 1 – 5 years
    2. 6 – 10 years
    3. ≥ 11 years

4. Comorbidities

    0. No
    1. Yes

5. Fasting blood glucose (FBG)……………………………… mg/dL

6. Glycated Hemoglobin (HbA1c)……………………………%

7. Medication adherence

    0. Non-adherence
    1. Daily adherence

8. Smoking status

    0. No
    1. Yes

9. Alcohol consumption

    0. No
    1. Yes

10. Dental service utilization

0. Never
1. Last visit > 1 year ago
3. At least once a year

11. Caregiver

0. No
1. Yes

1.1 Social Support Questionnaire

Instructions: Please place a check mark (✓) in one box only for each of the following statements, selecting the response that best reflects your actual situation, feelings, understanding, and opinions. There are no right or wrong answers.

Response Options

(1)	Yes	Definition	You agree with the statement.
(0)	Uncertain	Definition	You are uncertain about the statement.
(0)	No	Definition	You disagree with the statement.

Item	Statement	Yes	No	Uncertain
1.	You have someone who takes you to see dental personnel when you experience discomfort or problems in your mouth.			
2.	You have someone who takes you to see dental personnel at least once a year.			
3.	You have someone who helps provide you with oral hygiene supplies (e.g., toothpaste and a toothbrush).			
4.	You have someone who encourages you to participate in oral health care activities.			
5.	You have someone who provides you with advice when you have oral health problems.			
6.*	When you have a toothache, you have felt that you did not receive help or care from people around you or your caregiver.			
7.	You have someone who reminds you to avoid sweet, sticky, hard, and acidic foods that may damage your teeth.			
8.*	You have been teased or ignored when talking about your dental problems.			
9.	You receive printed or online educational materials about oral health.			
10.*	During the past 6 months, your caregiver has forgotten or neglected the importance of your dental appointments.			

Part 2: Oral Health Care Knowledge Questionnaire

Instructions: Please place a check mark (✓) in **one box only** that best represents your knowledge, understanding, opinions, and beliefs regarding the following statements. There are no right or wrong answers.

Response Options

(1)	Yes	Definition	You agree with the statement.
(0)	Do not know	Definition	You are uncertain about the statement.
(0)	No	Definition	You disagree with the statement.

Item	Statement	Yes	No	Do not know
1.	Periodontal disease can lead to tooth loss.			
2.	Bleeding gums are an early sign of gingivitis and periodontal disease.			
3.*	Smoking has no effect on gum and dental health.			
4.	Dry mouth in older adults is one of the causes of dental caries, gingivitis, and periodontal disease.			
5.*	Loose teeth or tooth decay are natural consequences of aging.			
6.	Brushing the teeth at least twice a day can prevent dental caries, gingivitis, and periodontal disease.			
7.	Avoiding food for 2 hours after brushing allows the anti-caries agents in toothpaste to work effectively.			
8.	If you experience bleeding after brushing your teeth, you should visit a dentist.			
9.*	Herbal toothpaste without fluoride can prevent dental caries.			
10.	Eating between meals, especially sugary and sticky foods, may cause dental caries, gingivitis, and periodontal disease.			

Part 3: Diabetes Knowledge Questionnaire

Instructions: Please place a check mark (✓) in **one box only** that best represents your knowledge, understanding, opinions, and beliefs regarding the following statements. There are no right or wrong answers.

Response Options

(1)	Yes	Definition	You agree with the statement.
(0)	Do not know	Definition	You are uncertain about the statement.
(0)	No	Definition	You disagree with the statement.

Item	Statement	Yes	No	Do not know
1.	Diabetes is a disease characterized by high blood glucose levels due to problems with the hormone insulin.			
2.	Older adults with diabetes are more likely to develop gum disease and dental caries than those without diabetes.			
3.	Oral wounds in people with diabetes may heal more slowly than those in people without diabetes.			
4.	People with diabetes often experience dry mouth, which may increase the risk of dental caries.			
5.*	Loose teeth are a normal problem in older adults and are not related to diabetes.			
6.	The dentist should be informed that the patient has diabetes and should know the patient's blood glucose level before performing tooth extraction or dental scaling.			
7.*	Older adults with diabetes should not undergo dental scaling because it can cause loose teeth.			
8.	Maintaining good oral health can help improve blood glucose control.			
9.	Dietary control and regular exercise not only help control blood glucose levels but also have beneficial effects on oral and dental health.			
10.	People with diabetes should have an oral health examination by a dentist at least once a year, even if they do not have a toothache.			

Part 4: Oral Health Care Behavior Questionnaire

Instructions: Please place a check mark (✓) in one box only for each of the following items, selecting the response that best reflects your actual oral health care practices or behaviors.

Response Options

(3)	Regularly	Definition	You perform the activity 5 or more days per week, or every time the relevant situation occurs.
(2)	Sometimes	Definition	You perform the activity less than 5 days per week.
(1)	Never	Definition	You never perform the activity.

Statement	Frequency of Practice		
	**Regularly**	**Sometimes**	**Never**
1.You use dental floss to clean between your teeth.			
2.You use mouthwash after brushing your teeth.			
3.You use toothpicks after meals.			
4.You use an interdental brush to clean between teeth where there are wider spaces.			
5.You choose a toothbrush with soft bristles, rounded bristle tips, and a brush head that is appropriately sized for your mouth.			
6.You spend more than 2 minutes brushing your teeth each time.			
7.You brush all surfaces of every tooth (outer, inner, and chewing surfaces).			
8.You brush your teeth using toothpaste that contains fluoride.			
9You brush your teeth in the morning after waking up and at night before going to bed.			
10.You brush your tongue every time after brushing your teeth.			
11.You brush your teeth after consuming sweets or sugary drinks (e.g., custard desserts, *khao tom mat* [sticky rice with banana], candies, or orange juice).			
12.You brush your teeth after eating sticky foods that tend to adhere to the teeth (e.g., *krayasat* [a Thai sweet made from grains, nuts, and syrup] or *khao niao daeng* [sweet red sticky rice]).			
13.You rinse your mouth with plain water immediately after eating.			
14.You avoid soft drinks and acidic foods or beverages that may cause tooth erosion.			
15.You avoid chewing hard foods (e.g., bones, roasted tamarind seeds, or grilled buffalo hide).			
